# Assessment of Consumer Complaint Investigation Scores, Recertification Survey Scores, and Overall Nursing Home Health Inspection Star Quality Rating

**DOI:** 10.1001/jamanetworkopen.2022.53952

**Published:** 2023-02-07

**Authors:** Lindsay J. Peterson, John R. Bowblis

**Affiliations:** 1 Florida Policy Exchange Center on Aging, School of Aging Studies, University of South Florida, Tampa; 2 Department of Economics and Scripps Gerontology Center, Miami University, Oxford, Ohio

## Abstract

**Importance:**

The Centers for Medicare & Medicaid Services’ Five-Star Quality Rating System combines results from nursing home recertification surveys and complaint investigations into a single indicator for health inspections. This combination may mask complaint investigation results.

**Objective:**

To construct and compare star ratings specific to recertification surveys and specific to complaint investigations to discern whether they provide different information.

**Design, Setting, and Participants:**

In this quality improvement study, the Nursing Home Compare Five-Star Quality Rating System was used to calculate three 5-star ratings: 1 overall health inspection rating combining recertification survey scores and complaint investigation scores, 1 using only recertification scores, and 1 using only complaint investigation scores. The study included US nursing homes. The sample calculated star ratings for nursing homes in November 2017. This sample included all whose most recent recertification surveys occurred in 2016 up to and including November 2017, and those with 36 months of data from the ASPEN Complaints/Incidents Tracking System and the Certification and Survey Provider Enhanced Reports. Data analyses were completed on different days in 2022, depending on which questions were being addressed.

**Main Outcomes and Measures:**

Comparison of the 3 star rating distributions. The recertification survey and complaint investigation star ratings were compared with respect to the overall health investigation rating. The recertification and complaint star ratings were cross-tabulated.

**Results:**

Among the 15 499 nursing homes, 19.8% had 1 overall health inspection star, 23.2% had 2, 23.2% had 3, 23.2% had 4, and 9.8% had 5 overall health inspection stars. Most had the same overall and recertification star ratings; for example, 79.4% had 5 overall stars and 5 recertification survey stars. However, overall and complaint-based star ratings were discordant, with a relatively large proportion of nursing homes (25.7%) having no complaint deficiencies and therefore high star ratings.

**Conclusions and Relevance:**

In this quality improvement study assessing the 2 components of the Five-Star Quality Rating System, results of recertification surveys were largely similar to health inspection star ratings. However, recertification survey scores differed from complaint inspection scores, suggesting health inspection ratings may not reflect consumers’ views of care, services, or other valued amenities. A complaint-focused metric may have utility. However, research is needed concerning the many nursing homes with no or very few complaint deficiencies.

## Key Points

**Question:**

Do results of nursing home recertification surveys and complaint investigations provide different information concerning nursing home quality?

**Findings:**

In this quality improvement study involving 15 499 US nursing homes, although data from recertification surveys and complaint investigations were combined into 1 health inspection star rating, differences were found between the 2 that the combined rating does not appear to reflect.

**Meaning:**

Results of this study suggest that a revision of the Centers for Medicare & Medicaid Services’ Five-Star Quality Rating System may be warranted to provide the public with information that more accurately reflects the perspectives of consumers.

## Teaser

This quality improvement study assesses star ratings that are specific to recertification surveys and ratings specific to complaint investigations for the purpose of assessing potential differences between the 2 components.

## Introduction

The Centers for Medicare & Medicaid Services (CMS) launched the Nursing Home Compare website in 1998 to provide high-quality information to help consumers “compare nursing homes more easily and identify areas about which [consumers] may want to ask questions.”^1^ As the complexity of the information increased, the CMS created the Five-Star Quality Rating System (Five-Star system) in 2008. The Five-Star system presents star ratings for each of 3 quality domains (health inspections, staffing, and quality measures) and a single overarching star rating.^2^ Each of the 4 ratings range from 1 (much below average) to 5 (much above average).^3^

The CMS Five-Star system is not without criticism, one of which concerns the reliability of information used to calculate star ratings. This rating is based on regulatory deficiencies issued during on-site recertification surveys and complaint investigations.^3,4^ Some nursing home stakeholders have taken issue with the predictability of these visits,^5,6^ which occur every 9 to 15 months, suggesting that results may not accurately reflect the quality of a nursing home that is able to address quality concerns before surveyors arrive.^5,6^ A recent report by The National Academies of Science, Engineering, and Medicine stated “the survey process often fails to properly identify serious care problems, fully correct and prevent recurrence of identified problems.”^7^

The focus of this study is the health inspection star rating, which is the foundation of a nursing home’s overall star rating.^3,8^ To calculate the health inspection rating, CMS uses the 3 most recent recertification surveys and all complaint investigations from the prior 36 months.^2^ Complaint investigations are distinct from recertification surveys because complaints can be made any time, and results may reflect conditions when the facility is not prepared for scrutiny. Notably, they originate with residents and nonresidents, including family members, nursing home staff, long-term care ombudsmen, and other concerned parties who may have a closer view of conditions than a surveyor on an annual visit. Researchers and policy analysts have proposed that consumer complaints provide a valuable source of quality information that differs from the information gathered through recertification surveys.^9,10,11,12,13^ Despite the specific nature of this information, the Five-Star system combines recertification survey and complaint investigation results into 1 overall health inspection star rating. Little is known about the extent to which complaints are reflected in the health inspection star rating. Therefore, in this study we constructed and compared star ratings that are specific to recertification surveys and ratings specific to complaint investigations for the purpose of assessing potential differences between the 2 components.

## Methods

### Data and Sample

The source of information on complaints is the ASPEN Complaints/Incidents Tracking System (ACTS). These data are based on information collected by state agencies as part of the federally required nursing home inspection process. The CMS requires states to track nursing home complaint investigations from filing through proposed action, including whether an investigation led to 1 or more deficiency citations for the nursing homes inspected.^4^ We merged the ACTS data with data from the Certification and Survey Provider Enhanced Reports system, which contains CMS data on each Medicare-certified nursing home (eg, number of beds, occupancy, profit status) and their recertification survey results. The study was reviewed by the University of South Florida institutional review board with a waiver of informed patient consent due to the research not involving human participants.

The analytic sample reflects star ratings as of November 2017, which required identifying the most recent recertification survey for all nursing homes that occurred in 2016 or 2017, leading up to November 2017. Furthermore, the sample was restricted to observations with 36 months of data, per the CMS star rating methods (N = 15 499).^2^ We used the November 2017 target because CMS revised federal nursing home regulations effective November 27, 2017, suspending calculation of health inspection star ratings for 1 year as state surveyors and nursing homes adjusted to the new regulations.^3^ The overall structure and intent of the regulations did not change, but regulations were expanded in some areas, such as staffing, as were guidelines to surveyors on how to interpret the regulations. Data analyses were completed on different days in 2022, depending on which questions were being addressed.

### Star Rating Calculations

As described in the Five-Star system technical users guide,^3^ CMS currently establishes the health inspection star rating by calculating a deficiency score using information from 2 sources, recertification surveys and complaint inspections. This score is calculated by assigning points to each regulatory deficiency from the prior 3 recertification surveys and complaint investigations from the prior 36 months. A deficiency reflects a surveyor’s observation that a nursing home is not in compliance with a federal regulation. Points assigned are based on the scope and severity of the deficiency identified. More recent deficiencies are assigned a greater weight in a deficiency score. This deficiency score is then compared with all other nursing homes in a state. Facilities in the top 10% are assigned 5 stars, the middle 70.0% are assigned 2, 3, or 4 stars (approximately 23.33% each), and the bottom 20.0% are assigned 1 star.

For the present study, we calculated 3 star ratings: an overall health inspection rating, which combines deficiencies from recertification surveys and complaint investigations and reflects the health inspection rating as now presented to consumers; a recertification health inspection rating, which uses only deficiencies issued from recertification surveys, and a complaint health inspection rating, which uses only deficiencies issued from complaint investigations. To assign star ratings, deficiency scores for each nursing home were determined. Scores did not include additional points CMS may assess if deficiencies are not addressed in a timely manner. Next, we determined state-specific cutoffs for each of the 3 star ratings. The cutoffs applied the CMS approach of assigning approximately 20% of nursing homes in a state a 1-star rating, 23.3% a 2-star, 3-star, and 4-star rating, and 10% a 5-star rating. More than 10% of a state’s nursing homes can be assigned a 5-star rating if a substantial number of nursing homes have no deficiencies.

### Statistical Analysis

Analyses were completed using SAS, version 9.4 (SAS Institute) and Stata, version 14 (StataCorp). First, we compared the distributions of the 3 star ratings we calculated. Next, to determine if the recertification and complaint health inspection star ratings varied in the information they contained, we compared these 2 with respect to the overall health inspection star rating. Specifically, we calculated the proportion of nursing homes whose recertification health inspection ratings were the same or different from the overall health inspection ratings and did the same for complaint health inspection ratings. Finally, we conducted a cross-tabulation of the recertification and complaint health inspection ratings. If the recertification surveys and complaint investigations provide similar information, most observations would be expected to have the same recertification and complaint health inspection star ratings. Conversely, if these 2 sources of quality data provide different information, we would expect to see variability in the observations.

## Results

The study included 15 499 nursing homes. Table 1 presents descriptive statistics. Facilities averaged 108 beds, 58.2% were part of a chain, and 59.1% primarily served Medicaid residents. Of the study nursing homes, the mean (SD) overall deficiency score was 44.7 (47.1) with most of the deficiency score coming from recertification deficiencies (42.5) compared with complaint deficiencies (2.3). A total of 25.7% of nursing homes had a 0 deficiency score for complaints.

**Table 1.** Summary Statistics^a^

Variable	Mean (SD)
Deficiency score	
Overall	44.7 (47.1)
Recertification	42.5 (45.6)
Complaint	2.3 (3.4)
Zero deficiency score facility, %	
Overall	0.7
Recertification	1
Complaint	25.7
No. of beds	108.4 (61.9)
Occupancy rate	79.4 (16.1)
Resident payer-mix	
Proportion Medicare	14.3 (14.6)
Proportion Medicaid	59.1 (20.0)
Type of facility, %	
For-profit ownership	69.7
Not-for-profit ownership	23.4
Hospital-based facility	4.9
Part of a chain	58.2

^a^ The sample includes 15 499 nursing homes. Facility characteristics are measured at the time of their most recent recertification inspection survey.

The Figure shows how these deficiency scores translated into the 3 star ratings we calculated. Across the 1-star through 5-star levels, the overall and recertification health inspection ratings were similarly distributed. However, the overall and complaint health inspection ratings were similar only at the lower star levels. Only half as many nursing homes received 4 stars based on complaint deficiency scores compared with those receiving 4 stars on overall and recertification deficiency scores, while the opposite was found at the 5-star level, where more than twice as many nursing homes received 5 stars based on complaint deficiency scores. These differences were evident even though the overall health inspection, recertification health inspection, and complaint health inspection ratings were constructed to have similar distributions. The high percentage of nursing homes with 5 complaint-based stars reflects the presence of nursing homes having no deficiencies associated with complaint investigations.

**Figure.**
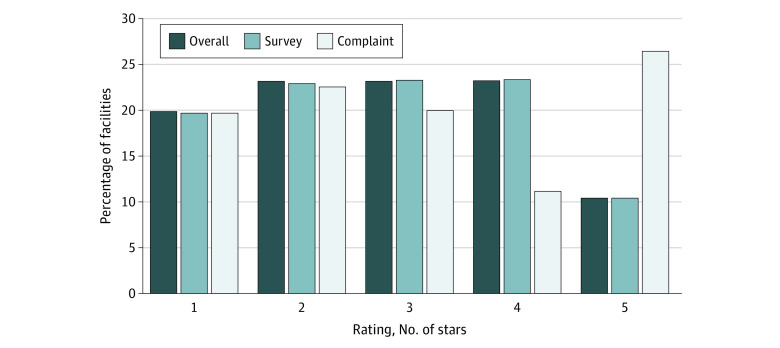
Comparison of Health Inspection Star Ratings, Overall, Recertification-Based, and Complaints-Based Frequency distribution of nursing home star ratings from 1 star (lowest) to 5 stars (highest) in 3 categories of health investigation results constructed for this study: overall (combining recertification survey and complaint investigation results), recertification survey alone, and complaint investigation alone.

Next, we examined recertification health inspection and complaint health inspection ratings, each with respect to the overall health inspection rating we constructed. Among the 15 499 nursing homes, 19.8%, had 1 overall health inspection star, 23.2%, had 2, 23.2% had 3, 23.2% had 4, and 9.8% had 5 overall health inspection stars. We found most nursing homes had the same overall and recertification health inspection ratings (Table 2). For instance, among the nursing homes with 5 stars based on their overall health inspection scores, 79.4% also received a 5-star recertification health inspection rating, and among the nursing homes with 4 overall health inspection stars, more than 70.0% also received 4 recertification health inspection stars. The variability was somewhat greater for nursing homes receiving 3 and 2 overall health inspection stars. However, in the analysis comparing the overall health inspection and complaint health inspection ratings, the variability was much greater (Table 3). Nursing homes with 1 star and 5 stars overall had the highest proportion of nursing homes with the same complaint health inspection rating (69.1% and 74.2%, respectively), but most of the nursing homes receiving 2, 3, or 4 stars from their overall health inspection scores had different complaint health inspection star ratings. For example, only 23.4% of nursing homes with 4 overall health inspection stars had 4 complaint health inspection stars. Table 4 displays the proportion of nursing homes with particular recertification and complaint health inspection star ratings. Only 30.9% of nursing homes are on the diagonal, meaning they had the same recertification and complaint health inspection star ratings. In other words, the vast majority of nursing homes (69.1%) had different recertification and complaint health inspection ratings. For example, a substantial majority of the 26.5% of nursing homes with 5 complaint health inspection stars had fewer recertification health inspection stars, reflecting the large number of nursing homes having no complaint deficiencies. However, a substantial proportion of nursing homes had fewer complaint health inspection stars than recertification health inspection star ratings. For example, among the 19.7% of nursing homes with 1 star for complaints, most had higher recertification health inspection ratings, meaning that their complaint investigation deficiency scores were worse than their recertification survey deficiency scores.

**Table 2.** Frequency of Recertification and Complaint Star Ratings Conditional on Overall Star Ratings

Overall star rating^a^	Recertification star rating, %^b^					No. of nursing homes (%)
	5	4	3	2	1	
5	79.4^c^	20.6	0	0	0	1627 (9.8)
4	6.6	70.0^c^	23.1	0.3	0	3603 (23.2)
3	1.4	14.0	55.8^c^	28.6	0.2	3600 (23.2)
2	0.9	4.9	15.9	55.4^c^	22.8	3602 (23.2)
1	0.6	2.8	6.4	17.3	72.9^c^	3067 (19.8)

^a^ Star ratings based on deficiencies found during recertification surveys and complaint investigations.

^b^ Star ratings based on deficiencies found during recertification surveys.

^c^ Percentage of nursing homes for which both measures agree.

**Table 3.** Frequency of Complaint Star Ratings Conditional on Overall Star Ratings

Overall star rating^a^	Complaint star rating, %^b^					No. of nursing homes (%)
	5	4	3	2	1	
5	69.1^c^	20.2	9.6	1.2	0	1627 (9.8)
4	42.4	22.0^c^	27.3	8.2	0.1	3603 (23.2)
3	24.2	11.5	34.1^c^	28.2	2.1	3600 (23.2)
2	12.6	4.6	17.2	46.1^c^	19.5	3602 (23.2)
1	4.1	1.1	4.2	16.3	74.2^c^	3067 (19.8)

^a^ Star ratings based on deficiencies found during recertification surveys and complaint investigations.

^b^ Star ratings based on deficiencies found during complaint investigations.

^c^ Percentage of nursing homes for which both measures agree.

**Table 4.** Comparison of Complaint Star Ratings and Recertification Star Ratings

Recertification star rating^a^	Complaint star rating, %^b^					% Total^c^
	5	4	3	2	1	
5	5.5^d^	1.8	1.8	1.1	0.4	10.6
4	8.6	3.7^d^	5.5	4.0	1.7	23.4
3	6.1	2.9	5.7^d^	5.6	3.1	23.3
2	4.3	2.0	4.9	6.7^d^	5.2	23.0
1	2.0	0.9	2.3	5.2	9.4^d^	19.8
% Total^c^	26.5	11.2	20.1	22.5	19.7	100

^a^ Star ratings based on deficiencies found during recertification surveys.

^b^ Star ratings based on deficiencies found during complaint investigations.

^c^ Numbers may not total 100 because of rounding.

^d^ Percentage of nursing homes for which both measures agree.

## Discussion

Research has reported that consumers may use the Five-Star system to make nursing home choices and that this system may motivate facilities to improve quality.^14^ The health inspection star rating is a key component of this system. It incorporates data from both recertification surveys and complaint investigations but is presented as a single rating. In this study, we broke the health inspection star rating into separate recertification and complaint ratings, doing so for 2 reasons. First, some stakeholders have claimed recertification surveys may not adequately reflect nursing home quality on a regular basis. Second, the Five-Star system does not include a specific measure of consumer satisfaction.

We found evidence that complaint investigations provide information that varies from information derived from recertification surveys. Specifically, the star ratings based on complaint investigations differed considerably from the overall health inspection star ratings we constructed. However, the overall health inspection star ratings were much more similar to recertification survey–based ratings. This finding suggests that overall health inspection star ratings may be affected by recertification survey results and may mask the results of complaint investigations. The US Government Accountability Office similarly observed a lesser role of complaints in a study about the utility of the star ratings to consumers trying to identify high-quality and low-quality nursing homes.^8^ It reported only a slight correlation (−0.27) between all complaints filed against nursing homes and a star rating constructed for the report that incorporated all domains (ie, health investigation, staffing, and quality measures). Our examination found evidence of alignment at the lowest star level. Many nursing homes that received only 1 complaint health inspection star by our calculations also received only 1 recertification health inspection star. However, we found a substantial proportion of nursing homes that received 1 complaint health inspection star but more, in some cases 2 or more, recertification health inspection stars, providing evidence that complaints have the potential to identify quality gaps not identified during recertification surveys.

With regard to nursing homes at the 5-star level, we found that a substantial number of nursing homes with 5-stars based on complaint investigations had fewer stars based on their recertification surveys. This finding is most likely the result of the number of nursing homes with no or very few substantiated complaints, which may or may not relate to quality. An absence of complaints may indicate that consumers are pleased with a nursing home’s care and service but also may indicate they are unaware of their complaint options, have difficulty reporting complaints, or fear retaliation. Long-term care ombudsmen have reported^15^ that racial and ethnic minority residents may fear retaliation and refrain from filing complaints. As another explanation for low numbers of substantiated complaints, it is possible that state survey agencies fail to substantiate some valid complaints because of a lack of evidence or the intake and/or investigation process was not well managed. Prior research has found wide variation by state in numbers of complaints filed and substantiated, with many states averaging fewer than 1 substantiated complaint per nursing home in a year’s time.^16^ Federal agency studies also have described gaps in the oversight and management of states’ nursing home complaints processes.^9,17,18,19^

The US Government Accountability Office recommended in 2016 that CMS evaluate the feasibility of adding consumer satisfaction survey results to the Five-Star system,^8^ in line with studies that have found satisfaction surveys measure unique dimensions of nursing home quality.^20,21^ Numerous surveys have been developed to measure nursing home satisfaction, though many are lengthy and/or not publicly available,^22^ with the exception of some states’ mandated surveys (eg, Minnesota, Ohio).^21,22,23^ As another way to augment the star ratings, a consumer-based measure of quality has been studied, with evidence indicating consumers place value on several nontechnical dimensions (eg, attentiveness to residents, physical environment) not captured in the Five-Star system ratings. The researchers suggested adding such information to Care Compare, formerly Nursing Home Compare.^24,25^ While CMS continues to revise the data provided to the public on nursing home quality, recently adding more detailed staffing data,^26^ it has demonstrated no clear progress toward adding consumer-focused quality data. The CMS responded to the US Government Accountability Office 2016 recommendation^8^ that while resident satisfaction information was important, collecting consistent data nationally was a challenge. The 2022 National Academies report discussed the need to include nursing home resident and family experience data in Care Compare, highlighting the federally developed Consumer Assessment of Healthcare Providers and Systems surveys. While emphasizing the importance of such data, the National Academies report also noted the challenges of conducting these surveys nationally.^7^

This challenge leaves the policy question of how the complaint process could be used to inform the public about quality concerns that may not be evident in recertification surveys. This question is important in light of recent proposals to survey high-performing nursing homes less frequently. The 2022 National Academies report stated that if such proposals came to pass, real-time quality metrics would be needed as an early warning system to monitor these facilities between surveys.^7^ Complaints could be the basis of such a warning system that additionally provided information about any nursing home’s quality trajectory. Research has found that complaints may signal the emergence of problems needing attention between survey visits.^11^ Complaints could additionally provide the public and regulators with valuable information when the regular survey process is interrupted, as during the COVID-19 pandemic.

Currently, CMS posts complaint investigations on Care Compare. An additional step could be to develop a complaint-focused quality metric, the benefits being that a system already exists for the collection of these data, and they do represent the perspectives of residents, families, and others with quality concerns; the drawbacks being that for nursing homes with no substantiated complaints it is unclear whether their consumers were pleased with care or faced barriers to lodging complaints and whether the complaints made were well investigated. Stevenson^11^ suggested the public would benefit from a metric including information about nursing homes’ total complaints, regardless of whether they resulted in deficiency citations. This suggestion addresses criticisms concerning the quality of complaint investigations and variation in survey processes between and within states. However, it does not address issues related to the low numbers of complaints filed.^15,16^ Presenting complaints data as currently collected poses the risk of sending the wrong messages, particularly concerning nursing homes with no or very few complaints. The use of consumer complaints data to better inform the public requires much more research concerning factors affecting the numbers of complaints lodged and how they are investigated, with the larger goal of making changes to ensure that (1) consumers understand they can anonymously lodge complaints directly or through their long-term care ombudsman, (2) complaint intake systems function in a way to reduce barriers to lodging complaints, and (3) complaints are fully investigated in a timely way.

### Limitations

This study has limitations. To our knowledge, no research has directly tested the validity of the ACTS data used for this analysis. However, those who conduct recertification surveys are also responsible for complaint surveys, and the system requests the same data from the states rather than states generating their own reports. In addition, the nature of the investigation process (eg, intake, interviews, and on-site visits), as described in the CMS State Operations Manual,^4^ substantiates the face validity of the ACTS data. As another limitation, our results are based on hypothetical star ratings. This basis was necessary because there are no existing data on nursing home star ratings based on recertification surveys or complaint investigations alone. The published details of the star rating methodology^3^ enabled us to break the overall health inspection score into its component scores. Additionally, we did not compare deficiencies issued during recertification surveys and those from complaint investigations, which was beyond the scope of this study. Finally, we scored only the complaint-based deficiencies generated through complaint investigations, not those handled during recertification surveys, which is the case when a complaint is made at a time near to a scheduled survey. This process means that some complaints were not included in the data used to construct complaint-based quality ratings. More research is warranted to examine whether there are differences between complaints that are investigated separately and those investigated as part of a survey.

## Conclusions

Overall, our findings substantially contribute to the research on nursing home complaints by suggesting that nursing home complaints can be a valuable source of information concerning nursing home quality. At the same time, the results highlight critical questions about the complaints process, particularly concerning the number of nursing homes with no or very few complaints. It is possible that more robust complaints filing and investigation processes could substantially enhance the role complaints play in the Five-Star system and further improve the utility of complaints as indicators of nursing home quality.

## Notes

Supplement.
Data Sharing Statement
