# Supplementary material for: Assessment of Consumer Complaint Investigation Scores, Recertification Survey Scores, and Overall Nursing Home Health Inspection Star Quality Rating
Source: JAMA Netw Open. 2023 Feb 7;6(2):e2253952. doi: 10.1001/jamanetworkopen.2022.53952 (PMC10408269; doi:10.1001/jamanetworkopen.2022.53952)
Supplement: Supplement. — Data Sharing Statement [file jamanetwopen-e2253952-s001.pdf]

Data Sharing Statement

Peterson. Assessment of Consumer Complaint Investigation Scores, Recertification Survey Scores, and Overall Nursing Home Health Inspection Star Quality Rating. *JAMA Netw Open*. Published February 07, 2023. doi:10.1001/jamanetworkopen.2022.53952

Data
Data available: Yes
Data types: Data (not involving human participants)
How to access data: https://data.cms.gov/provider-data/topics/nursing-homes/data-sources
When available: With publication

Supporting Documents
Document types: None

Additional Information
Who can access the data: This data is publicly available.
Types of analyses: The data is available for any purpose.
Mechanisms of data availability: This data is publicly available.
